# Accurate and efficient representation of intra­molecular energy in *ab initio* generation of crystal structures. I. Adaptive local approximate models

**DOI:** 10.1107/S2052520616015122

**Published:** 2016-12-01

**Authors:** Isaac Sugden, Claire S. Adjiman, Constantinos C. Pantelides

**Affiliations:** a Molecular Systems Engineering Group Centre for Process Systems Engineering Department of Chemical Engineering, Imperial College London, London SW7 2AZ, England

**Keywords:** crystal structure prediction, solid-state science, local apprioximate model

## Abstract

The global search stage of crystal structure prediction (CSP) methods requires a fine balance between accuracy and computational cost, particularly for the study of large flexible molecules. A major improvement in the accuracy and cost of the intramolecular energy function used in the *CrystalPredictor II* [Habgood *et al.* (2015 ▸). *J. Chem. Theory Comput.* **11**, 1957–1969] program is presented, where the most efficient use of computational effort is ensured *via* the use of adaptive local approximate model (LAM) placement. The entire search space of the relevant molecule’s conformations is initially evaluated using a coarse, low accuracy grid. Additional LAM points are then placed at appropriate points determined *via* an automated process, aiming to minimize the computational effort expended in high-energy regions whilst maximizing the accuracy in low-energy regions. As the size, complexity and flexibility of molecules increase, the reduction in computational cost becomes marked. This improvement is illustrated with energy calculations for benzoic acid and the ROY molecule, and a CSP study of molecule (XXVI) from the sixth blind test [Reilly *et al.* (2016 ▸). *Acta Cryst.* B**72**, 439–459], which is challenging due to its size and flexibility. Its known experimental form is successfully predicted as the global minimum. The computational cost of the study is tractable without the need to make unphysical simplifying assumptions.

## Abstract 2

This article describes an important improvement in the *CrystalPredictor II* code: adaptive Local Approximate Models (LAMs). This improvement allows the most efficient use of computational effort to cover a flexible molecule’s conformational space, and is illustrated with a crystal structure prediction (CSP) investigation into the sixth blind test molecule 26.

## 1. Introduction   

The primary aim of crystal structure prediction (CSP) techniques is to produce a ranked list of all the potential crystal structures for a molecule or set of molecules. Because of the significant effect that crystal structure has on solid-state properties, such as colour, solubility and hygroscopicity, such a ranked list offers a wealth of information and many opportunities to improve the development of new crystalline materials (Price *et al.*, 2016 ▸; Neumann *et al.*, 2015 ▸). In the case of the pharmaceutical industry, the appearance of a new or unexpected form or polymorph can have major legal and economic ramifications, particularly if solubility/bioavailability are affected, as illustrated by the cases of the appearance of Ritonavir form II (Chemburkar *et al.*, 2000 ▸) and the Zantac litigation (Seddon, 1999 ▸). Furthermore, the ability to tune a molecule’s solid-state properties through predictive approaches would be very useful to industries that rely on crystalline materials. Therefore, significant benefits are offered by the possibility of predicting a molecule’s crystal structure(s), especially when this is possible *via ab initio* techniques that rely only on molecular structure information.

Whilst a relatively new field, CSP methods for organic molecules have undergone considerable improvements over the past few years, as seen in the increasing size and complexity of molecular targets in the blind tests organized by the Cambridge Crystallographic Data Centre, as well as the increasing level of success achieved in the tests (Day *et al.*, 2005 ▸, 2009 ▸; Bardwell *et al.*, 2011 ▸; Motherwell *et al.*, 2002 ▸; Lommerse *et al.*, 2000 ▸). The targets that CSP groups are being asked to investigate as a matter of routine are becoming more industrially relevant, with larger, more flexible molecules that could be seen as drug analogues now being considered. Indeed, in the case of molecule (XXIII) in the sixth blind test (Reilly *et al.*, 2016 ▸), the target represents a former drug candidate for the treatment of Alzheimer’s disease. All the targets were chosen so as to present challenges that test the theories and computational capabilities currently available.

### 1.1. Global search in CSP   

The central tenet of CSP is that the crystal structures that are most likely to form will be low-energy minima on the free-energy surface, with respect to structural variables, namely: cell lengths and angles, the molecular position and orientation, and the molecule’s internal degrees of freedom (Pantelides *et al.*, 2014 ▸; Brandenburg & Grimme, 2014 ▸; Woodley & Catlow, 2008 ▸; Price, 2008 ▸; Cruz-Cabeza *et al.*, 2015 ▸). Thermodynamically, the most stable crystal structure (at given temperature and pressure) is the global minimum on the Gibbs free-energy surface; however, given the cost inherent in free-energy calculations and the comparatively small energetic contributions arising from entropic effects, most CSP methods use lattice energy/enthalpy rather than free energy in order to rank the predicted crystal structures.

A major factor in successfully identifying all likely polymorphs is the trade-off made between the accuracy of the model used to describe the differences in energy between a molecule’s possible structures (often less than 5 kJ mol^−1^), and the extent of the search for low-energy minima across the entire free-energy surface. In view of this, most CSP techniques use a broadly two-stage methodology: a first-stage global search that is used to search for low-energy structures on the lattice energy surface using a relatively low-cost, less accurate lattice energy model; and a second-stage refinement that takes the most promising structures from the first stage and re-ranks them *via* local energy minimization, using a more accurate and computationally demanding lattice energy model. All the successful predictions in the sixth blind test (Reilly *et al.*, 2016 ▸) used some variant of this multi-stage methodology.

In order to identify all potential low-energy polymorphs, the first stage must perform an extensive search (typically involving hundred of thousands of points) of the lattice energy surface over sufficiently wide ranges of the lattice energy model variables (cell lengths and angles, conformational degrees of freedom *etc.*); therefore, the efficiency of the lattice energy model is very important. Moreover, since only a relatively small proportion (typically a few hundreds) of the lowest-energy structures identified will be passed for refinement to the second stage, the lattice energy model employed by the first stage also needs to be sufficiently accurate not to exclude any potential polymorphs from further consideration.

Overall, achieving the right trade-off between the efficiency and accuracy of the first-stage lattice energy model is a key challenge for CSP. If the accuracy of the lattice energy model can be increased at moderate computational cost, the risk of missing low-energy structures can be decreased. Furthermore, the cost of the second stage can be reduced significantly as increased confidence in the ranked list of structures generated in the first stage typically allows a decrease in the number of structures that must be taken through to the computationally intensive refinement stage, opening the possibility for the latter to employ even higher-accuracy lattice energy models.

This paper focuses on significantly improving the efficiency of the global search stage *via* improvements to the *CrystalPredictor* algorithm (Karamertzanis & Pantelides, 2007 ▸, 2005 ▸; Habgood *et al.*, 2015 ▸), which has been used extensively in blind tests and in a variety of CSP applications (see, for example, Bardwell *et al.*, 2011 ▸; Day *et al.*, 2009 ▸; Braun *et al.*, 2013 ▸, 2014 ▸, 2016 ▸; Vasileiadis *et al.*, 2012 ▸; Eddleston *et al.*, 2015 ▸; Uzoh *et al.*, 2012 ▸).

Before describing specific advances, we give a brief overview of the algorithm to the extent necessary for the purposes of this paper.

### 1.2. The *CrystalPredictor* algorithm   

The *CrystalPredictor* algorithm (Karamertzanis & Pantelides, 2007 ▸, 2005 ▸; Habgood *et al.*, 2015 ▸) is a global search algorithm based on a large number of gradient-based local minimizations starting from crystal structures generated by a Sobol sequence (Sobol, 1967 ▸), a low-discrepancy technique that ensures the best coverage of the space of the variables that uniquely define a crystal structure.

The original version of the algorithm *CrystalPredictor I* (Karamertzanis & Pantelides, 2007 ▸, 2005 ▸) was used successfully in several CSP studies to produce initial ranked lists of crystal structures. However, in order to ensure that all experimentally known structures are identified by the CSP, it was often found to be necessary to refine the 1000 to 1500 lowest-energy structures in these initial lists, which resulted in very significant computational costs (see, for example, Vasileiadis *et al.*, 2012 ▸, 2015 ▸).

The above issue with *CrystalPredictor I* was partly caused by the insufficiently accurate description of the effects of molecular conformation on both the intramolecular and intermolecular contributions to the lattice energy. This realisation led to an improved version of the algorithm, *CrystalPredictor II* (Habgood *et al.*, 2015 ▸), using a more accurate energy function that utilizes local approximate models (LAMs; Kazantsev *et al.*, 2010 ▸). LAMs allow the efficient and accurate calculation of intramolecular energy as a function of flexible torsion angles (‘independent’ conformational degrees of freedom, 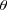). Moreover, LAMs also allow the values of those degrees of freedom that are not explicitly treated as flexible in the minimization (the ‘dependent’ degrees of freedom, 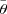, including bond lengths, bond angles and any torsion angles that are not included in 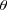) to be determined as functions of the independent conformational degrees of freedom, 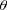.

*CrystalPredictor* assumes that the lattice energy of a crystal is given as a function of the cell lengths and angles, collectively denoted as *X*, as well as the positions and orientations of the molecules in the asymmetric unit, collectively denoted as β, and the molecules’ independent conformational degrees of freedom, 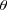. The optimization then seeks to minimize a lattice energy function, 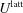 of the form
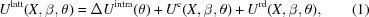
where the intermolecular energy is separated into (*a*) an electrostatic term, 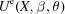, evaluated by the Coulombic attraction between atom centres, based on point charges obtained using isolated molecule *ab initio* calculations, and (*b*) a repulsion/dispersion term, 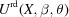, described by Buckingham potentials whose parameters have been fitted to experimental data, typically the FIT potential (Cox *et al.*, 1981 ▸; Williams, 1984 ▸; Coombes *et al.*, 1996 ▸). We note that both 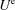 and 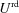 are functions of molecular conformation 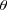 as it affects intermolecular distances. In general, the electronic charge distribution within the molecule is also a function of molecular conformation, and therefore the atomic charges used in 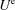 may also depend on 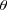.

The intramolecular energy contribution, 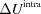 is given by
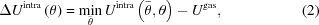
where 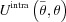 is the intramolecular energy of an isolated molecule at conformation 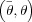 and 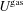 is the minimum energy of the unconstrained isolated molecule (*i.e. in vacuo*, with all internal degrees of freedom allowed to vary). To avoid expensive repeated *ab initio* calculations for the evaluation of the terms 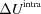 and 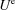 during the global search, a set of reference calculations at values of the 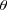 on a regular grid are performed before the start of the global search, and are subsequently used in *CrystalPredictor* to obtain a low-cost approximation of these energies at any point. The two versions of *CrystalPredictor* differ in how the approximation is constructed. In *CrystalPredictor II*, the intramolecular energy at some value 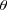 of the independent conformational degrees of freedom is calculated from the LAM with the closest matching conformation, 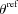, on the grid using an approximation of the form
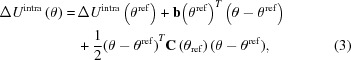
whilst the set of dependent degrees of freedom 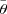 is obtained by a linear approximation of the form
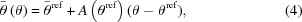
where the matrices ***A*** and ***C*** and the vector ***b*** are given by (Kazantsev *et al.*, 2011 ▸)
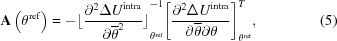


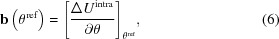


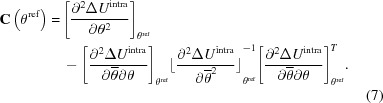
The variation of point charges with conformation is neglected in *CrystalPredictor II*, so that the point charges used to evaluate 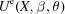 are taken as those at 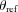, *i.e.* 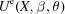 = 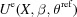.

The global search domain in terms of independent conformational degrees of freedom can be denoted as 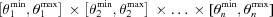, where *n* is the total number of independent conformational degrees of freedom and 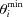 and 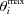, 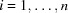, are selected to include all areas of practical interest, typically where 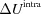 is below 20 to 30 kJ mol^−1^. LAMs are calculated at grid points whose location depends on the size of the search domain and a user-specified grid spacing 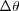. The conformational space is therefore partitioned into hyper-rectangles of the form
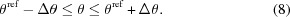
The LAM validity range, 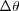, needs to be small enough to ensure that expressions (3) and (4) provide sufficiently good approximations for the intramolecular energy and dependent degrees of freedom within a certain conformational distance from 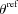.

The adoption of a regular LAM grid has been found to be effective in CSP for several molecules, such as β-d-glucose, ROY and a pharmaceutical compound, BMS-488043 (Habgood *et al.*, 2015 ▸). However, the number of LAM points needed to achieve a desired coverage grows exponentially with the number of degrees of freedom. For highly flexible molecules, where the number of independent conformational degrees of freedom is large, and the range of flexibility, [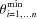, 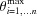], to be searched is wide, the number of LAMs to be calculated incurs a high computational cost. In such cases, the choice of an appropriate 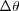 has a significant impact on both the accuracy and computational cost, and its determination requires substantial analysis of the molecule of interest prior to computing the grid points.

### 1.3. Aims   

In this paper we propose improvements to the algorithm that address the issues identified above, leading to reduced computational cost and improved accuracy. In particular, we seek to achieve this by introducing an adaptive LAM implementation so that the LAM points no longer need to be placed on a regular grid.

A motivating example for the development of an improved algorithm is introduced in §2, based on molecule (XXVI) from the sixth blind test (Reilly *et al.*, 2016 ▸). The adaptive LAM placement algorithm is described in §3, and the reduction in computational cost that it offers is analysed. Finally, in §4, we revisit the motivating example, applying to it the improved *CrystalPredictor II* algorithm in the context of a complete CSP study of molecule (XXVI) from the sixth blind test.

## 2. Motivating example: molecule (XXVI) of the sixth blind test   

**Figure 1:**
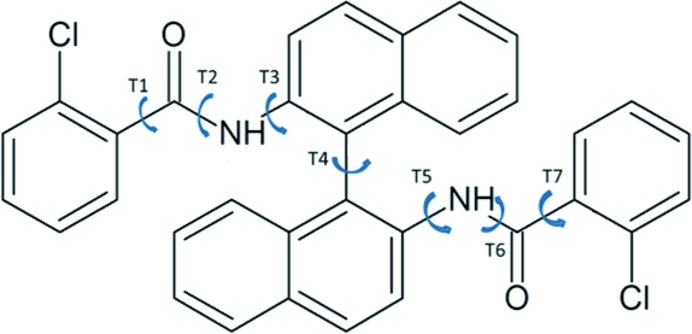
Molecular diagram of molecule (XXVI) and independent degrees of freedom.

The recent blind test on crystal structure prediction methods, organized by the Cambridge Crystallographic Data Centre, sought to evaluate the capabilities of current computational methods in predicting the crystal structures of organic molecules. Five targets were chosen, representing challenges to the crystal structure prediction community. The two versions of *CrystalPredictor* were deployed by two of the participating groups, in combination with *CrystalOptimizer*, to identify *Z*′ = 1 structures. This approach resulted in the identification of the known experimental structures within the predicted energy landscapes in most cases. However, in the case of molecule (XXVI), shown in Fig. 1 ▸, the multiple flexible torsion angles present particular difficulties, which are discussed here and motivate the development of an improved version of *CrystalPredictor II*.

Molecule (XXVI) contains the common 1,1′-binaphthalene fragment, which can feature axial chirality, although no chiral precursors were present in the synthesis. As reported by Reilly *et al.* (2016 ▸), there are currently two known pure experimental forms: form (1) is a *Z*′ = 1 structure crystallized in the 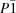 space group, while form (11) is a structure discovered through polymorph screening by Johnson Matthey (Pharmorphix), after the conclusion of the blind test, for which no structural data are currently available. In addition, there are nine reported solvates. Unusually for 1,1′-binaphthalene-based molecules, one O atom is unsatisfied in terms of hydrogen bonds, and the angles and torsions in the amide group and phenyl rings are somewhat outside expected ranges. This is a result of the bulkiness of the 1,1′-binaphthalene and phenyl groups, as well as the internal hydrogen bond occurring between the chlorine in one half of the molecule, and the amide group in the opposite half. The number and unusual values of the independent degrees of freedom, as well as its sheer size, contribute to the difficulties posed by this molecule.

In the sixth blind test (Reilly *et al.*, 2016 ▸), the use of *CrystalPredictor I* and *CrystalOptimizer* by the Price *et al.* group successfully led to the identification of form (1) as the lowest energy structure in the final landscape. The use of *CrystalPredictor I*, however, required making severe assumptions on flexibility to limit the computational cost; as is usually done with *CrystalPredictor I* when there are many flexible degrees of freedom, the flexible torsion angles were divided into three groups (group 1 containing *T*1, group 2 containing *T*3–5, and group 3 containing* T*7). This approach has been successful in other investigations (Vasileiadis *et al.*, 2012 ▸) and relies on the assumption that flexible torsions in distinct parts of the molecule can rotate independently, with their effect on 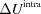 being largely unaffected by the values of the flexible torsions in the other torsion groups. However, in the case of molecule (XXVI), since the benzene groups that rotate in the different halves of the molecule are in close proximity to each other, such an assumption may not be fully justified in this case. The loss of accuracy arising from this treatment was acknowledged by the Price *et al.* team and was countered by applying the second-stage refinement to a wider than usual range of the low-energy crystal structures predicted in the *CrystalPredictor I* landscape. More specifically, a single iteration of *CrystalOptimizer* was applied to each of the 9400 structures identified by *CrystalPredictor I* within 40 kJ mol^−1^ of the global minimum, thereby resulting in re-ranking of the structures. The full *CrystalOptimizer* calculation was then performed for the 1322 lowest-energy structures. Although in this case the experimental form was successfully identified, this decomposition approach is not generally applicable to all molecules, and a more accurate method of covering the conformational space is needed (see Habgood *et al.*, 2015 ▸) for a more complete discussion).

Our research group’s submission for molecule (XXVI) made use of *CrystalPredictor II*, with all seven torsional angles shown in Fig. 1 ▸ being treated as independent degrees of freedom, 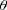. The domain of each angle that was deemed to be relevant for CSP purposes was initially decided by analysis of crystal structures in the Cambridge Structure Database (CSD) and the results of one-dimensional scans through each independent degree of freedom. A LAM validity range 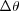 of ±15° was used for all torsional angles, resulting in a grid comprising 2592 LAM points (see Table 1 ▸). The computation of the latter at the HF/6-31G(d,p) level of theory required approximately 200 000 CPU h [typically on Intel(R) Xeon(R) CPU E5-2660 v2 running at 2.20 GHz].

Our normal practice in the applications of *CrystalPredictor II* is to also evaluate the intramolecular energies at the edges of the search space; if these are found to be lower than a user-specified threshold (typically 20–30 kJ mol^−1^), the search space is expanded. In the case of molecule (XXVI), this investigation identified energies lower than 10 kJ mol^−1^ on the boundaries of the domains for torsions T3 and T5, and therefore these domains would normally have to be expanded quite significantly. However, a larger regular grid with the domain of the two key torsions extended by the necessary 120° would involve 11 858 LAMs, and their construction would require approximately 910 000 CPU h. As this was impracticable within the time constraints of the blind test, it was decided not to extend the search beyond the domains indicated in Table 1 ▸.

As indicated in Table 1 ▸, the experimental value for the torsion angle T5 is 222.46°, which unfortunately lies outside the search domain [95°, 185°]. As a result, our search failed to identify form (1) of molecule (XXVI). This illustrates the importance of developing new techniques that would allow large conformational spaces to be covered efficiently by LAMs, which in turn provides the motivation for the present work.

## 3. An algorithm for adaptive LAM placement   

This section presents an adaptive algorithm that automatically positions LAMs at points in the search domain of the independent degrees of freedom, where necessary to ensure the required degree of accuracy. Firstly, the revised algorithm for generating new LAMs is summarized in §3.1, with examples of its implementation given in §§3.2 and 3.3.

### 3.1. Adaptive generation of LAMs   

The basic idea of the adaptive LAM placement algorithm proposed in this paper is to take an existing set of LAMs placed over the search domain of the independent conformational degrees of freedom 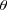, and to try to identify a point at which these LAMs may not attain the required accuracy. If such a point is found, then a new LAM is then generated at that point. The procedure is repeated until no new point is found to be necessary.

Establishing the exact error of the approximation provided by a LAM at a particular point 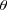 would require performing the corresponding quantum mechanical calculation and comparing its results with the LAM predictions. As this would defeat the purpose of an efficient LAM placement algorithm, we choose to use an approximate criterion based on the difference in the predictions at a point 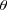 between two neighbouring LAMs.

In particular, we assume that the maximum discrepancy between the predictions of two LAMs generated at points 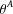. and 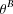. respectively, is likely to occur around the mid-point 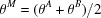. Using equation (3), we can then easily compute the quantities 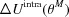 using the two LAMs. If we denote these by 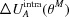 and 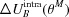, then a new LAM is generated at point 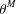 only if these quantities differ by more than a certain specified threshold, 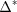, in absolute value, *i.e.*

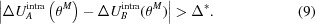
However, before deciding whether to generate a new LAM at *M*, there are two additional conditions we need to consider. First, it is unnecessary to generate a LAM at point *M* if the latter is unlikely to be inside the region which would be relevant for the purposes of CSP, *i.e.* if 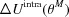 exceeds a given threshold, 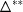. Of course, the exact value of 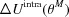 is not known, but it can be approximated by the values obtained by the two LAMs. Conservatively, we choose to consider the lower of these two values; therefore, another necessary criterion for a LAM to be generated at point *M* is
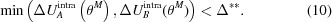
A second consideration that needs to be taken into account is that the above reasoning is valid only if the LAMs *A* and *B* are indeed those nearest to point *M*. If there exists a third LAM *C* which is nearer to *M* than either *A* or *B*, then of course the accuracy of the approximations provided by the LAMs at *A* and *B* at point *M* is irrelevant: neither of those would be used during the search to determine the quantity 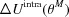. Therefore, a third necessary criterion for a LAM to be generated at point *M* is
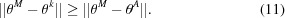
For each and every existing LAM *k* other than *A* and *B*, where the norm 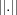 is the Euclidean norm in conformational space.

The above ideas provide the basis of the new adaptive algorithm for LAM generation. Given any set of LAMs, we consider each and every pair (*A*, *B*), determine its midpoint *M*, and test criteria (9)–(11) [Disp-formula fd10] [Disp-formula fd11]. If all of those are found to be true, then a new LAM is generated at point *M*, and the procedure is repeated until no more new LAMs are found to be necessary.

In our current implementation, the algorithmic parameters 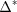 and 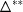 are set by default at 1 and 20 kJ mol^−1^, respectively. Using a smaller value of 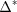 leads to increased consistency between LAMs, but also results in the addition of a greater number of LAM points and hence higher computational cost. We have found the value of 1 kJ mol^−1^ to give an appropriate balance between cost and consistency. The default value of 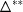 is chosen based on the assessment of Thompson & Day (2014 ▸) of the maximum energetic cost of molecular distortion away from gas phase conformation in naturally occurring polymorphs. Here, using a larger value of 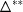 increases the reliability of the LAMs for higher-energy conformations, but this again comes at the cost of adding more LAMs. The norm in criterion (11) is based on the Euclidean distance 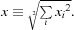
The initial set of LAMs is constructed over a relatively coarse regular grid which is then subsequently refined according to the algorithm presented here, resulting in a complete set of LAMs prior to the start of the global search. A flowchart describing this process is provided in the supporting information. As in the previous implementation of *CrystalPredictor II* (Habgood *et al.*, 2015 ▸), during the search, equations (3) and (4) are applied using the LAM that is nearest, in the Euclidean distance sense, to the current point 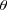.

### 3.2. Illustrative example 1: benzoic acid   

**Figure 2:**
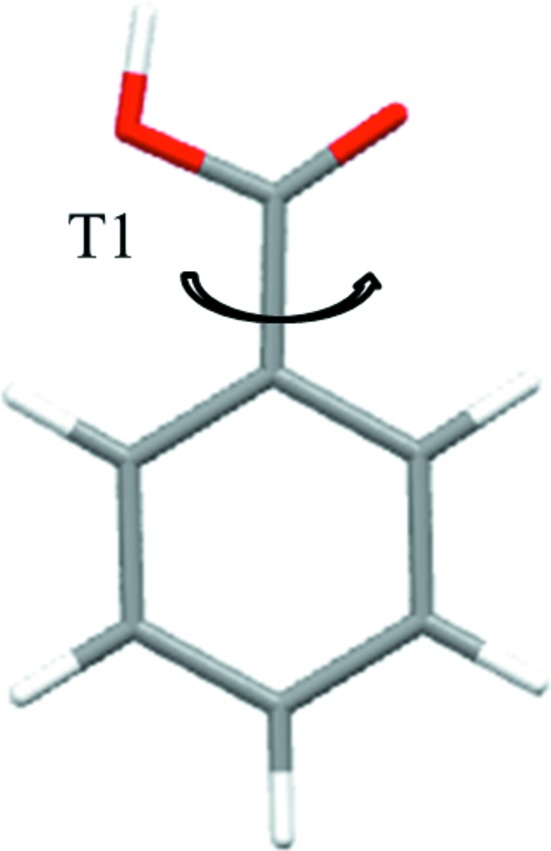
Molecular diagram and degree of freedom *T*1 for benzoic acid, at *T*1 = 0.

In order to better understand the concept of adaptive LAM placement, we first consider a molecule with a single independent degree of freedom, namely benzoic acid (see Fig. 2 ▸). The chemically relevant domain for torsion angle *T*1 is initially covered by four LAMs based at the points *T*1 = −90, −30, +30 and +90°, at the M06/6-31G(d,p) level of theory.

**Figure 3:**
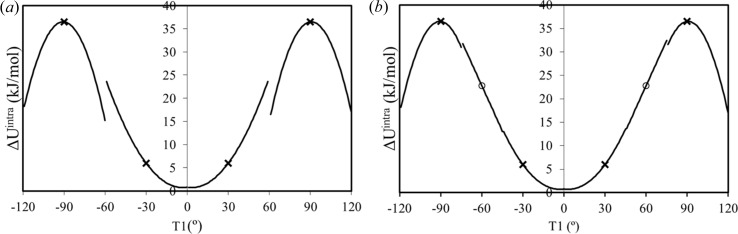
Intramolecular energy for benzoic acid based on one-dimensional LAMs. (*a*) Initial regular grid and (*b*) final LAM placement. Crosses represent LAMs on the initial regular grid, circles LAMs added to eliminate mismatch between adjacent LAMs.

As can be seen in Fig. 3 ▸(*a*), there is clearly a significant mismatch (9.2 kJ mol^−1^) in the intramolecular energy contribution predicted by adjacent LAMs at *T*1 = ± 60°. This can be corrected by inserting two LAMS at these positions, as illustrated in Fig. 3 ▸(*b*). On the other hand, there is no such mismatch at the boundary between the original second and third LAMs at *T*1 = 0°, and therefore no new LAM needs to be inserted there. This consistency check, in which different LAM predictions are compared to each other, ensures that the intramolecular energy is described consistently by the LAMs at the given boundary. It does not, however, guarantee that that *ab initio* accuracy is achieved, although we note that LAMs have been shown to represent *ab initio* results very well in their locality (Kazantsev *et al.*, 2011 ▸). In the case of a symmetric molecule such as benzoic acid, the consistency of the LAMs at *T*1 = 0° could be attributed to the symmetric placement of LAM points and does not imply agreement with the *ab initio* energy value. This could be addressed through the manual addition of LAMs by the user to break symmetry where appropriate.

Overall, achieving the same level of accuracy with a regular grid would require a grid spacing of 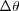 = ± 15°, *i.e.* 7 LAMs overall (starting with one based at *T*1 = −90°), as opposed to the 6 LAMs shown in Fig. 3 ▸(*b*). Whilst only a small saving is achievable in this simple case, much more marked efficiencies can be achieved for molecules involving multiple independent degrees of freedom, as illustrated by the next example.

### 3.3. Illustrative example 2: the ROY molecule   

**Figure 4:**
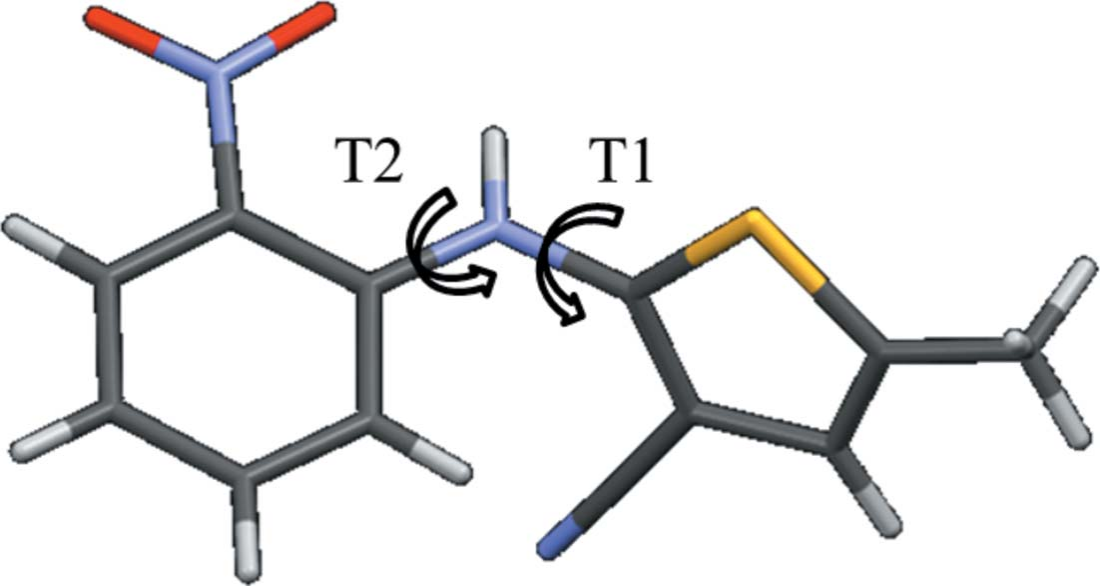
Molecular diagram and the two independent conformational degrees of freedom considered for 5-methyl-2-[(2-nitrophenyl)­amino]-3-thiophenecarbonitrile (ROY).

The adaptive algorithm is further illustrated for the ROY molecule (5-methyl-2-[(2-nitrophenyl)­amino]-3-thiophenecarbonitrile) (Yu, 2010 ▸), which is considered here to involve two independent conformational degrees of freedom, *T*1 and *T*2, as shown in Fig. 4 ▸. These two degrees of freedom have broad ranges of flexibility, with *T*1 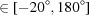 and T2 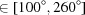, but within this overall conformational space there are large regions that are characterized by high intramolecular energy which are unlikely to be of relevance to CSP.

**Figure 5:**
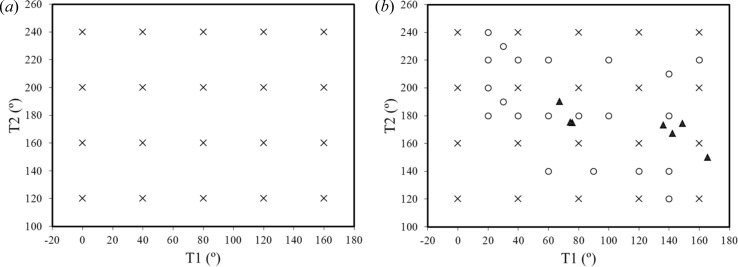
LAM placements for ROY. (*a*) Initial regular grid (20 LAMs), with 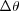 = ±20°. (*b*) Final LAM set (41 LAMs) derived by adaptive LAM placement algorithm. Crosses represent LAMs in the initial regular grid, circles LAMs added by adaptive placement algorithm; triangles show the positions of experimentally known conformations.

Starting with an initial uniform grid generated with 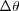 = ±20° and comprising 20 LAMs, at the B3LYP/6-31G(d,p) level of theory, the application of the LAM generation algorithm results in the final set of 41 LAMs shown in Fig. 5 ▸(*b*). The minimum spacing between these LAMs is 14°; a regular grid constructed over the original domain would require about 163 LAMs to achieve the same minimum spacing (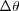 ≃ ±7°). However, many of these LAMs would be unnecessary: for example, we note that the adaptive algorithm does not introduce any new LAM points in the region 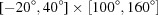. Fig. 5 ▸(*b*) also shows the positions of the six known experimental forms of ROY (Yu, 2010 ▸). This demonstrates that the algorithm does indeed focus computational effort on relevant areas of conformational space.

**Figure 6:**
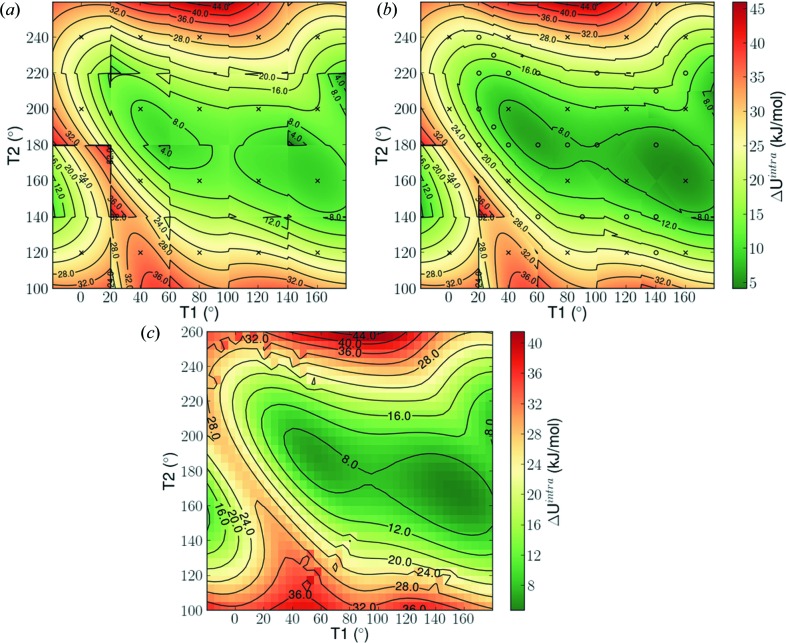
Intramolecular energy (in kJ mol^−1^) as predicted by LAMs in 0.5° scan across conformational space; (*a*) under a regular coarse grid (Δθ = ±20°), (*b*) using the adaptive LAM placement of Fig. 5 ▸(*b*). Crosses represent regular LAMs, circles non-uniform/adaptive LAMs and (*c*) *Ab initio* intramolecular energy based on a 5° scan.

The intramolecular energy predictions by the original and final sets of LAMs are shown in Figs. 6 ▸(*a*) and (*b*), respectively. It is clear that the low conformational energy regions are not rectangular, *i.e.* there is significant interaction between the two torsional angles. It can also be seen that the adaptive LAM placement leads to a smoother intramolecular energy surface in these key regions.

**Figure 7:**
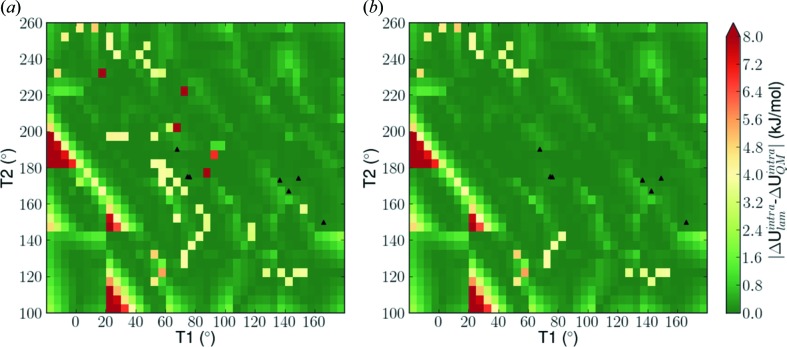
Absolute difference between *ab initio* and LAM predicted intramolecular energies (kJ mol^−1^) based on a 5° scan, with LAMs computed based on (*a*) the coarse regular grid of Fig. 5 ▸(*a*). (*b*) The adaptive scheme of Fig. 5 ▸(*b*). The black triangles represent experimental conformations.

The intramolecular energy contribution is also computed *ab initio* over the same range of degrees of freedom at 5° increments and shown in Fig. 6 ▸(*c*). Visual comparison of the three energy landscapes show that key qualitative features are captured by both LAM-based approximations. A more quantitative comparison is presented in Figs. 7 ▸(*a*) and (*b*), where the differences between the LAM approximation and the *ab initio* energies are computed at 5° intervals. The average absolute deviation for the regular coarse grid scheme is 0.75 kJ mol^−1^, while for the adaptive scheme it is 0.56 kJ mol^−1^. More importantly, it is evident that with the regular grid, there are many areas in which the error is more than 5 kJ mol^−1^, particularly at the edges of LAM validity. This can lead to the generation of a low-accuracy energy landscape during the global search, in which some structures are found to have unrealistically low or high lattice energy. Finally, it can be seen that in the areas surrounding the experimental structures (black triangles), improved accuracy is achieved.

## 4. CSP investigation of molecule (XXVI)   

The proposed algorithm is now applied to molecule (XXVI) from the sixth blind test (*cf*. §2). As shown in Fig. 1 ▸, the molecule has 7 flexible degrees of freedom, several of which have broad ranges of flexibility.

In this new CSP study, the search domains for all 7 torsion angles are extended until the intramolecular energies 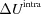 at the edges of the search space exceed 15 kJ mol^−1^. While a larger cutoff value has been used in previous work (Habgood *et al.*, 2015 ▸), 15 kJ mol^−1^ is a practical value given the computational cost, and the low likelihood of a molecular distortion with an energetic cost greater than 15 kJ mol^−1^ (Thompson & Day, 2014 ▸). As can be seen by a comparison of the search domains listed in Tables 1 ▸ and 2 ▸ this now results in much wider domains for *T*3 and *T*5 than those used in our original CSP study (Reilly *et al.*, 2016 ▸).

### 4.1. Generation of an appropriate LAM set   

In applying the algorithm proposed in this paper, we start with a relatively coarse regular grid with increments of 60° for *T*1, *T*3, *T*5 and *T*7, and 30° for *T*2, *T*4 and *T*6 (see Table 2 ▸), again, at the HF/6-31G(d,p) level of theory. Overall, this initial grid requires the generation of 1152 LAMs. We then apply the adaptive LAM placement algorithm of §3 in a single-pass mode, *i.e.* simply considering all pairs of points in the initial grid and deciding whether to place a LAM at their mid-point. Overall, this results in the generation of an additional 2491 LAMs.

**Figure 8:**
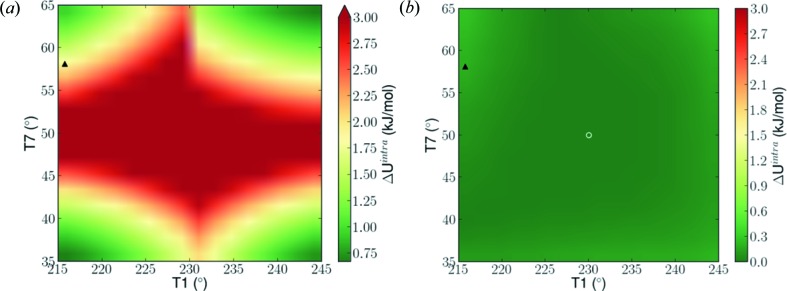
Absolute error between *ab initio* and LAM predicted energies for the experimental form (1), across the validity range of the nearest LAM point on the adaptive grid to the experimental values of *T*1 and *T*7, calculated at 2° increments. The filled triangle indicates the experimental values of *T*1 and *T*7. (*a*) Error obtained using the initial LAM set constructed on a regular grid; the four LAMs used for these calculations are outside the domain shown at (200°, 80°), (200°, 20°), (260°, 20°) and (260°, 20°), for *T*1 and *T*7, respectively. (*b*) Error obtained using the final LAM set, including a new LAM point indicated by an open white circle.

The accuracy gain achieved by the judicious placement of new LAM points is illustrated in Fig. 8 ▸ for a 30° × 30° sub-region near the experimental values of torsions *T*1 and *T*7 (indicated by a filled triangle). The figures show the differences between the value of 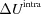 predicted using the nearest LAM and the corresponding *ab initio* value. The underlying data are generated by varying *T*1 and *T*7 in 2° increments, while keeping the other 5 torsional angles constant at the values *T*2 = 180.0°, *T*3 = 170.0°, *T*4 = 70.0°, *T*5 = 230.0° and *T*6 = 180.0°.

Fig. 8 ▸(*a*) shows results obtained using the initial LAM set on a regular grid. The four nearest LAMs used for this purpose are outside the domain shown. As can be seen, the values of 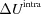 involve non-negligible errors, with a maximum of 5.15 kJ mol^−1^ across the sub-region and a value of 1.01 kJ mol^−1^ at the experimental values of *T*1 and *T*7. On the other hand, Fig. 8 ▸(*b*) shows results obtained with the final LAM set which now includes a new LAM placed at the position indicated by the open circle. It can clearly be seen that the addition of this single new point in this sub-region results in very substantial reduction in the error in 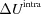. The maximum error across this sub-region is now 0.27 kJ mol^−1^, with the error at the experimental values of *T*1 and *T*7 being just 0.09 kJ mol^−1^.

As has already been noted in §2, a regular grid that would cover the entire domain of interest at the required accuracy would have to incorporate 11 858 LAMs, whose construction would require approximately 910 000 CPU h on the computing hardware used for this study. Instead, the LAM set determined by the new adaptive LAM placement algorithm requires only 3643 LAMs, an overall reduction of just under 70%.

### 4.2. Global search using *CrystalPredictor II*   

**Figure 9:**
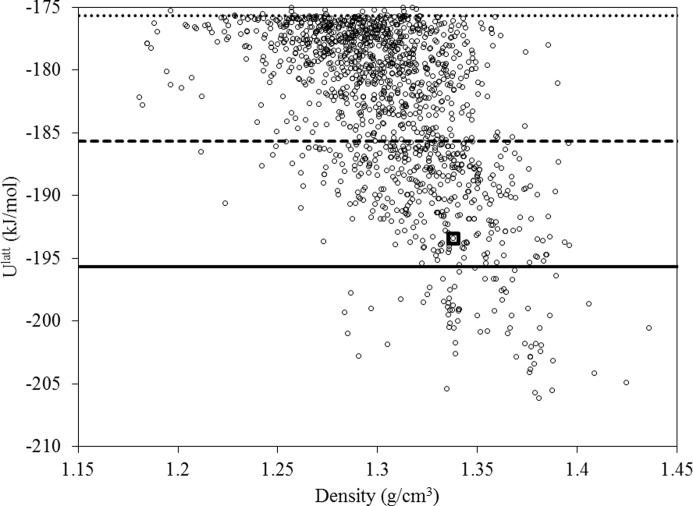
*CrystalPredictor II* energy landscape for molecule (XXVI) based on 1000 000 minimizations and adaptive LAM placement. The square denotes the experimental form, the solid line is the 10 kJ mol^−1^ cut-off from the global minimum, and the heavy and light dashed lines are the 20 and 30 kJ mol^−1^ cut-offs from the global minimum, respectively.

A global search over 1 000 000 candidate structures is performed using *CrystalPredictor II*, making use of the LAM set determined above. As shown in Fig. 9 ▸, this results in 81 unique structures being identified within 10 kJ mol^−1^ of the global minimum, with 465 and 1413 unique structures being identified within, respectively, 20 and 30 kJ mol^−1^. The experimental form is identified as the 130th lowest energy structure, with a lattice energy 12.27 kJ mol^−1^ greater than the global minimum, and a good reproduction of the experimental geometry (RMSD_20_ = 0.595 Å).

### 4.3. Refinement of low-energy crystal structures using *CrystalOptimizer*   

**Figure 10:**
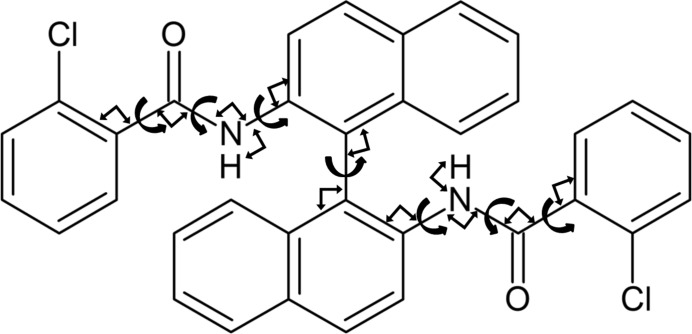
Independent conformational degrees of freedom used in the *CrystalOptimizer* investigation of molecule (XXVI). Curly arrows represent torsions, block arrows represent angles.

*CrystalOptimizer* minimizations are performed on the 1413 unique structures that were identified within 30 kJ mol^−1^ from the global minimum (*cf*. Fig. 9 ▸). The approach followed was identical to that in our original investigation carried out in the context of the sixth blind test (see supporting information in the blind test paper by Reilly *et al.* (2016 ▸). In particular, intramolecular energy and conformational multipoles were determined using quantum mechanical calculations at the PBE1PBE 6-31G(d,p) level of theory, and an extended set of independent conformational degrees of freedom was considered as seen in Fig. 10 ▸. The use of a different level of theory from *CrystalPredictor* implies that it is not possible to re-use the LAMs generated at the global search stage. If the same level of theory were used, this would result in a reduction of the number of quantum mechanical calculations at the refinement stage.

**Figure 11:**
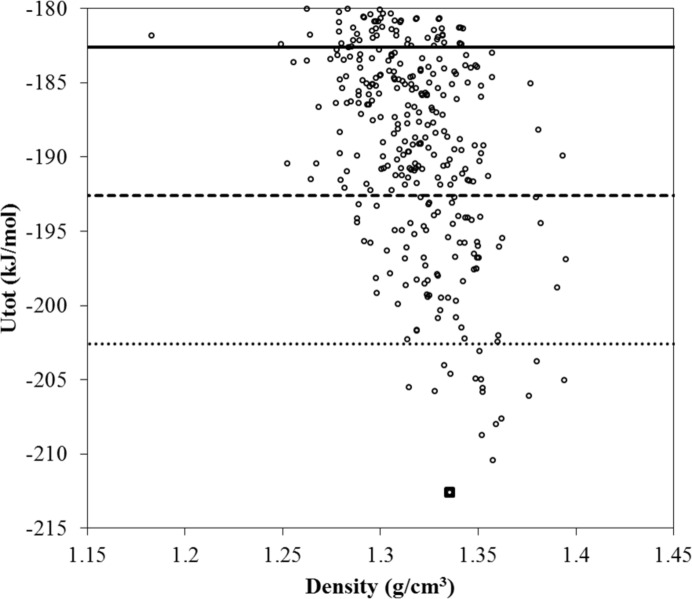
Lattice energy landscape following *CrystalOptimizer* results for molecule (XXVI). The structures generated following the refinement of the 1413 structures generated by the global search stage within 30 kJ mol^−1^ of the lowest-energy structure are shown. The lowest 100 unique structures span a lattice energy range of 20.8 kJ mol^−1^ from the global minimum, whilst only 17 unique structures are identified with lattice energies within 10 kJ mol^−1^ of the global minimum. The square denotes the experimental form, the solid line is the 10 kJ mol^−1^ cut-off from the global minimum, and the heavy and light dashed lines are the 20 and 30 kJ mol^−1^ cut-offs from the global minimum, respectively.

**Figure 12:**
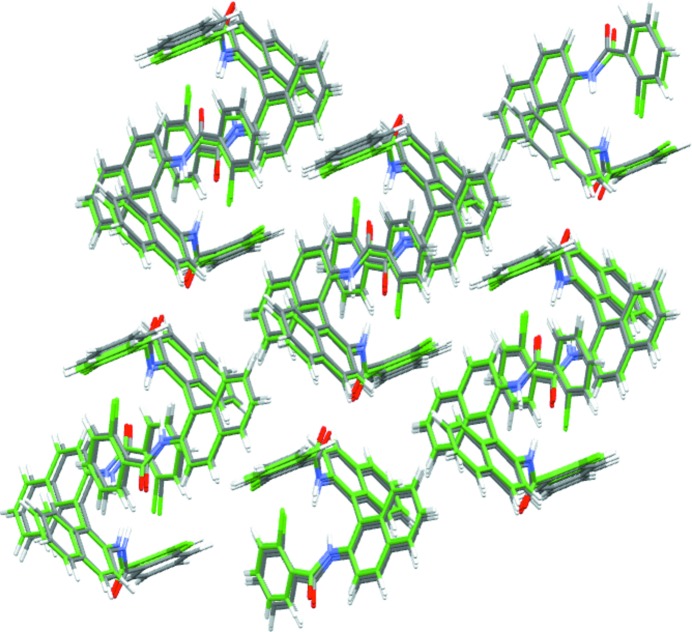
Overlay of the global minimum predicted structure (green tubes) generated in the *CrystalOptimizer* energy landscape, and the experimental structure (grey tubes = C atoms, red = O, blue = N, white = H).

The resulting energy landscape is presented in Fig. 11 ▸. The experimental form is found at the global minimum, with another 17 structures having lattice energy within 10 kJ mol^−1^ from the global minimum, and 92 within 20 kJ mol^−1^. We note that these numbers are significantly lower than the corresponding numbers of structures determined at the end of the global search (81 and 465, respectively); thus, refinement using a more accurate lattice energy model and taking account of a higher degree of conformational flexibility has resulted in substantial clarification of the polymorphic landscape. We also note that the geometry of the experimental structure is reproduced with good accuracy (RMSD_20_ = 0.330 Å), as illustrated in Fig. 12 ▸ and Table 3 ▸.

The computational cost of the CSP study is summarized in Table 4 ▸. The generation of LAM points remains the most significant cost but is now tractable given the high-dimensionality of this molecule.

## 5. Concluding remarks   

The 2016 blind test (Reilly *et al.*, 2016 ▸) revealed that achieving an appropriate balance between computational cost and accuracy in the global search for crystal structures remains a challenge for large molecules. The algorithm presented in this paper addresses this issue by introducing the adaptive placement of LAMs within the *CrystalPredictor II* algorithm, an improvement on the uniform grid scheme which had proved too computationally demanding to apply to molecule (XXVI). A higher density of LAM points is automatically achieved in chemically interesting areas of conformational space, thereby resulting in a more efficient use of expensive *ab initio* calculations. This, in turn, allows the *CrystalPredictor II* algorithm to handle larger molecules and to explore larger areas of conformational space, through an effective global search methodology. The successful application of this new approach to molecule (XXVI) realises one of the aims of the blind tests, namely to drive innovation in CSP by providing unique and challenging molecular systems.

Throughout the *CrystalPredictor II* calculations, the lattice energy for any given molecular conformation 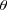 is computed by making use of the LAM that is nearest to 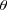. One undesirable effect of this approach is that discontinuities in both the lattice energy and its partial derivatives may occur at points 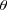 that lie on the boundaries between adjacent LAMs. Such discontinuities may cause numerical difficulties for CrystalPredictor’s gradient-based optimization algorithm in cases in which the path of the optimization iterations crosses one or more LAM boundaries. In practical terms, this is usually exhibited by the algorithm reaching a point from which it cannot achieve any further reduction in lattice energy, despite the fact that the mathematical optimality conditions are not yet strictly satisfied. In the calculations reported in this paper and in our previous work, we have chosen to adopt a conservative approach whereby such points are still considered as candidates for further refinement. However, this may result in much additional computation: for example, in the case of molecule (XXVI), 1283 of the 1413 structures that underwent final refinement (*cf*. §4.3) actually belonged to this category. Part II of this paper (Sugden & Adjiman, 2016 ▸) is concerned with addressing this problem in a more fundamental manner by removing the discontinuities at the LAM boundaries.

Data statement: Data underlying this article can be accessed on Zenodo at https://doi.org/10.5281/zenodo.56731, and used under the Creative Commons Attribution licence.

## Supplementary Material

Crystal structure: contains datablock(s) 1, 2, 3, 4, 5, 6, 7, 8, 9, 10, 11, 12, 13, 14, 15, 16, 17, 18, 19, 20, 21, 22, 23, 24, 25, 26, 27, 28, 29, 30, 31, 32, 33, 34, 35, 36, 37, 38, 39, 40, 41, 42, 43, 44, 45, 46, 47, 48, 49, 50, 51, 52, 53, 54, 55, 56, 57, 58, 59, 60, 61, 62, 63, 64, 65, 66, 67, 68, 69, 70, 71, 72, 73, 74, 75, 76, 77, 78, 79, 80, 81, 82, 83, 84, 85, 86, 87, 88, 89, 90, 91, 92, 93, 94, 95, 96, 97, 98, 99, 100, 101, 102, 103, 104, 105, 106, 107, 108, 109, 110, 111, 112, 113, 114, 115, 116, 117, 118, 119, 120, 121, 122, 123, 124, 125, 126, 127, 128, 129, 130, 131, 132, 133, 134, 135, 136, 137, 138, 139, 140, 141, 142, 143, 144, 145, 146, 147, 148, 149, 150, 151, 152, 153, 154, 155, 156, 157, 158, 159, 160, 161, 162, 163, 164, 165, 166, 167, 168, 169, 170, 171, 172, 173, 174, 175, 176, 177, 178, 179, 180, 181, 182, 183, 184, 185, 186, 187, 188, 189, 190, 191, 192, 193, 194, 195, 196, 197, 198, 199, 200, 201, 202, 203, 204, 205, 206, 207, 208, 209, 210, 211, 212, 213, 214, 215, 216, 217, 218, 219, 220, 221, 222, 223, 224, 225, 226, 227, 228, 229, 230, 231, 232, 233, 234, 235, 236, 237, 238, 239, 240, 241, 242, 243, 244, 245, 246, 247, 248, 249, 250, 251, 252, 253, 254, 255, 256, 257, 258, 259, 260, 261, 262, 263, 264, 265, 266, 267, 268, 269, 270, 271, 272, 273, 274, 275, 276, 277, 278, 279, 280, 281, 282, 283, 284, 285, 286, 287, 288, 289, 290, 291, 292, 293, 294, 295, 296, 297, 298, 299, 300, 301, 302, 303, 304, 305, 306, 307, 308, 309, 310, 311, 312, 313, 314, 315, 316, 317, 318, 319, 320, 321, 322. DOI: 10.1107/S2052520616015122/wf5128sup1.cif

Further developments. DOI: 10.1107/S2052520616015122/wf5128sup2.pdf

CCDC references: 1506361, 1506362, 1506363, 1506364, 1506365, 1506366, 1506367, 1506368, 1506369, 1506370, 1506371, 1506372, 1506373, 1506374, 1506375, 1506376, 1506377, 1506378, 1506379, 1506380, 1506381, 1506382, 1506383, 1506384, 1506385, 1506386, 1506387, 1506388, 1506389, 1506390, 1506391, 1506392, 1506393, 1506394, 1506395, 1506396, 1506397, 1506398, 1506399, 1506400, 1506401, 1506402, 1506403, 1506404, 1506405, 1506406, 1506407, 1506408, 1506409, 1506410, 1506411, 1506412, 1506413, 1506414, 1506415, 1506416, 1506417, 1506418, 1506419, 1506420, 1506421, 1506422, 1506423, 1506424, 1506425, 1506426, 1506427, 1506428, 1506429, 1506430, 1506431, 1506432, 1506433, 1506434, 1506435, 1506436, 1506437, 1506438, 1506439, 1506440, 1506441, 1506442, 1506443, 1506444, 1506445, 1506446, 1506447, 1506448, 1506449, 1506450, 1506451, 1506452, 1506453, 1506454, 1506455, 1506456, 1506457, 1506458, 1506459, 1506460, 1506461, 1506462, 1506463, 1506464, 1506465, 1506466, 1506467, 1506468, 1506469, 1506470, 1506471, 1506472, 1506473, 1506474, 1506475, 1506476, 1506477, 1506478, 1506479, 1506480, 1506481, 1506482, 1506483, 1506484, 1506485, 1506486, 1506487, 1506488, 1506489, 1506490, 1506491, 1506492, 1506493, 1506494, 1506495, 1506496, 1506497, 1506498, 1506499, 1506500, 1506501, 1506502, 1506503, 1506504, 1506505, 1506506, 1506507, 1506508, 1506509, 1506510, 1506511, 1506512, 1506513, 1506514, 1506515, 1506516, 1506517, 1506518, 1506519, 1506520, 1506521, 1506522, 1506523, 1506524, 1506525, 1506526, 1506527, 1506528, 1506529, 1506530, 1506531, 1506532, 1506533, 1506534, 1506535, 1506536, 1506537, 1506538, 1506539, 1506540, 1506541, 1506542, 1506543, 1506544, 1506545, 1506546, 1506547, 1506548, 1506549, 1506550, 1506551, 1506552, 1506553, 1506554, 1506555, 1506556, 1506557, 1506558, 1506559, 1506560, 1506561, 1506562, 1506563, 1506564, 1506565, 1506566, 1506567, 1506568, 1506569, 1506570, 1506571, 1506572, 1506573, 1506574, 1506575, 1506576, 1506577, 1506578, 1506579, 1506580, 1506581, 1506582, 1506583, 1506584, 1506585, 1506586, 1506587, 1506588, 1506589, 1506590, 1506591, 1506592, 1506593, 1506594, 1506595, 1506596, 1506597, 1506598, 1506599, 1506600, 1506601, 1506602, 1506603, 1506604, 1506605, 1506606, 1506607, 1506608, 1506609, 1506610, 1506611, 1506612, 1506613, 1506614, 1506615, 1506616, 1506617, 1506618, 1506619, 1506620, 1506621, 1506622, 1506623, 1506624, 1506625, 1506626, 1506627, 1506628, 1506629, 1506630, 1506631, 1506632, 1506633, 1506634, 1506635, 1506636, 1506637, 1506638, 1506639, 1506640, 1506641, 1506642, 1506643, 1506644, 1506645, 1506646, 1506647, 1506648, 1506649, 1506650, 1506651, 1506652, 1506653, 1506654, 1506655, 1506656, 1506657, 1506658, 1506659, 1506660, 1506661, 1506662, 1506663, 1506664, 1506665, 1506666, 1506667, 1506668, 1506669, 1506670, 1506671, 1506672, 1506673, 1506674, 1506675, 1506676, 1506677, 1506678, 1506679, 1506680, 1506681, 1506682

## Figures and Tables

**Table 1:** Independent conformational degrees of freedom considered for molecule (XXVI) by our group during the sixth blind test The experimental values are the reported values of the torsions in form (1) (Reilly *et al.*, 2016 ▸) and were not available to us during the blind test. The bold experimental value indicates that this torsion lies outside the specified search space.

Independent degree of	LAM regular grid			Experimental value in form (1) (°)
freedom, 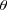 (*cf*. Fig. 1 ▸)	Search domain (°)	Spacing Δθ (°)	No. of grid points	(not available during the blind test)
*T*1	[0, 360]	±15	12	215.76
*T*2	[165, 195]	±15	1	181.26
*T*3	[95, 185]	±15	3	163.34
*T*4	[55, 115]	±15	2	78.47
*T*5	[95, 185]	±15	3	**222.46**
*T*6	[165, 195]	±15	1	185.06
*T*7	[0, 360]	±15	12	301.872

**Table 2:** Search domain and initial LAM grid for CSP study on molecule (XXVI)

Independent degree of	Initial LAM grid			Experimental value in form (1) (°)
freedom, 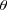 (*cf*. Fig. 1 ▸)	Search domain (°)	Spacing Δθ (°)	No. of grid points	(not available during the blind test)
*T*1	[0, 360]	±30	6	215.76
*T*2	[165, 195]	±15	1	181.26
*T*3	[20, 260]	±30	4	163.34
*T*4	[55, 115]	±15	2	78.47
*T*5	[20, 260]	±30	4	222.46
*T*6	[165, 195]	±15	1	185.06
*T*7	[0, 360]	±30	6	301.872

**Table 3:** Structural information for the predicted crystal structure for molecule (XXVI)

Molecule (XXVI)	ρ (g cm^−3^)	*a* (Å)	*b* (Å)	*c* (Å)	α (°)	β (°)	γ (°)	Rank	*U* ^latt^ (kJ mol^−1^)	RMSD_20_ (Å)
Experimental	1.332	10.40	11.03	14.18	76.83	73.33	63.47	–	–	–
*CrystalPredictor II*	1.332	10.23	10.74	14.95	89.14	72.42	64.02	130	−193.41	0.60
*CrystalOptimizer*	1.337	10.31	11.25	14.10	79.81	73.97	62.88	1	−212.59	0.33

**Table 4:** Computational cost of CSP for molecule (XXVI)

Step	No. of calculations	CPU h (approximate)
Step 0: construction of LAM regular grid	3643	280 000
Step 1: *CrystalPredictor II* minimizations	1 000 000	20 000
Step 2: *CrystalOptimizer* refinements	1413	80 000
Total	–	380 000
