# Supplementary material for: Accurate and efficient representation of intra­molecular energy in ab initio generation of crystal structures. I. Adaptive local approximate models
Source: Acta Crystallogr B Struct Sci Cryst Eng Mater. 2016 Dec 1;72(Pt 6):864–74. doi: 10.1107/S2052520616015122 (PMC5134761; doi:10.1107/S2052520616015122)
Supplement: Supplementary file 2 [file b-72-00864-sup2.pdf]

STRUCTURAL SCIENCE
CRYSTAL ENGINEERING
MATERIALS

Volume 72 (2016)

Supporting information for article:

Accurate and efficient representation of intramolecular energy in *ab initio* generation of crystal structures. Part I: Adaptive local approximate models

Isaac Sugden, Claire S. Adjiman and Constantinos C. Pantelides

Further development since the last release of CrystalPredictor2: New density check

Once the LAM points have been generated, the computational efficiency of the global search itself is an important success factor. While it is desirable to investigate potential structures across a wide space of possibilities, the automatic generation of initial structures can lead to physically-unrealistic configurations. In all versions of CrystalPredictor, a starting structure is obtained by generating a Sobol' point in the space of structural variables, such as cell lengths and angles, given lower and upper bounds on these variables. Given the broad range of cell lengths seen in organic crystal structures, a practical range for a cell length is [4Å, 80Å]. The large width of this range has meant that, in previous versions of CrystalPredictor, many starting structures have an unsuitably low density and were therefore rejected before a minimisation was attempted or had a high density and a corresponding high energy, leading to failed or computationally expensive minimisations. In the version presented here, a new scheme is introduced: the vector of Sobol' variables that define an initial structure are extended to include a density variable, ρ (in g/cm^3), with a range that is defined by the user. This variable defines the density of the unit cell for the initial structure being generated. The cell lengths generated by the Sobol' sequence are rescaled to match the density structural variable, keeping their ratios constant. Thus, an initial structure is derived as follows:

1. Generate Sobol' point, including cell lengths; *a', b', c',* cell angles; *α, β,* and *γ* and density, *ρ.*
2. Calculate scaling factor, *S* such that $\rho = f(a'S, b'S, c'S, \alpha, \beta, \gamma, Z', N, m)$, where N is the number of molecules in the unit cell (determined by space group), Z' is determined by the user before the run and *m* is the mass of the molecule.
3. Set the cell lengths of the initial structure as $a = a'S$, $b = b'S$, $c = c'S$

Choosing appropriate bounds for density ensures that the structural space is covered, but there is a significantly better chance of generating physically-realistic starting structures, leading to a more efficient searching stage, as illustrated for the example of Molecule XXIII in H An approximate doubling of the rate is observed in this case, although some experimenting with different density ranges may be necessary with each new molecule, to see this improvement in all cases.

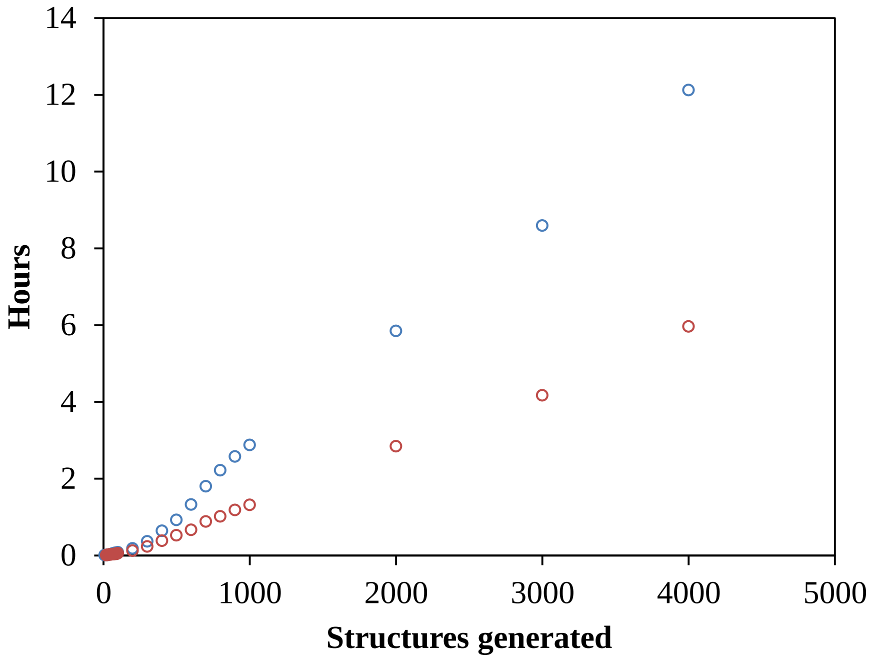

Figure S1 Hours to complete structures for Molecule XXIII under different schemes. Red circles represent CrystalPredictor under the density constraint scheme (0.9-1.3 g/cm^3), blue circles under the old scheme.

Flowchart to describe the adaptive generation of LAMs algorithm

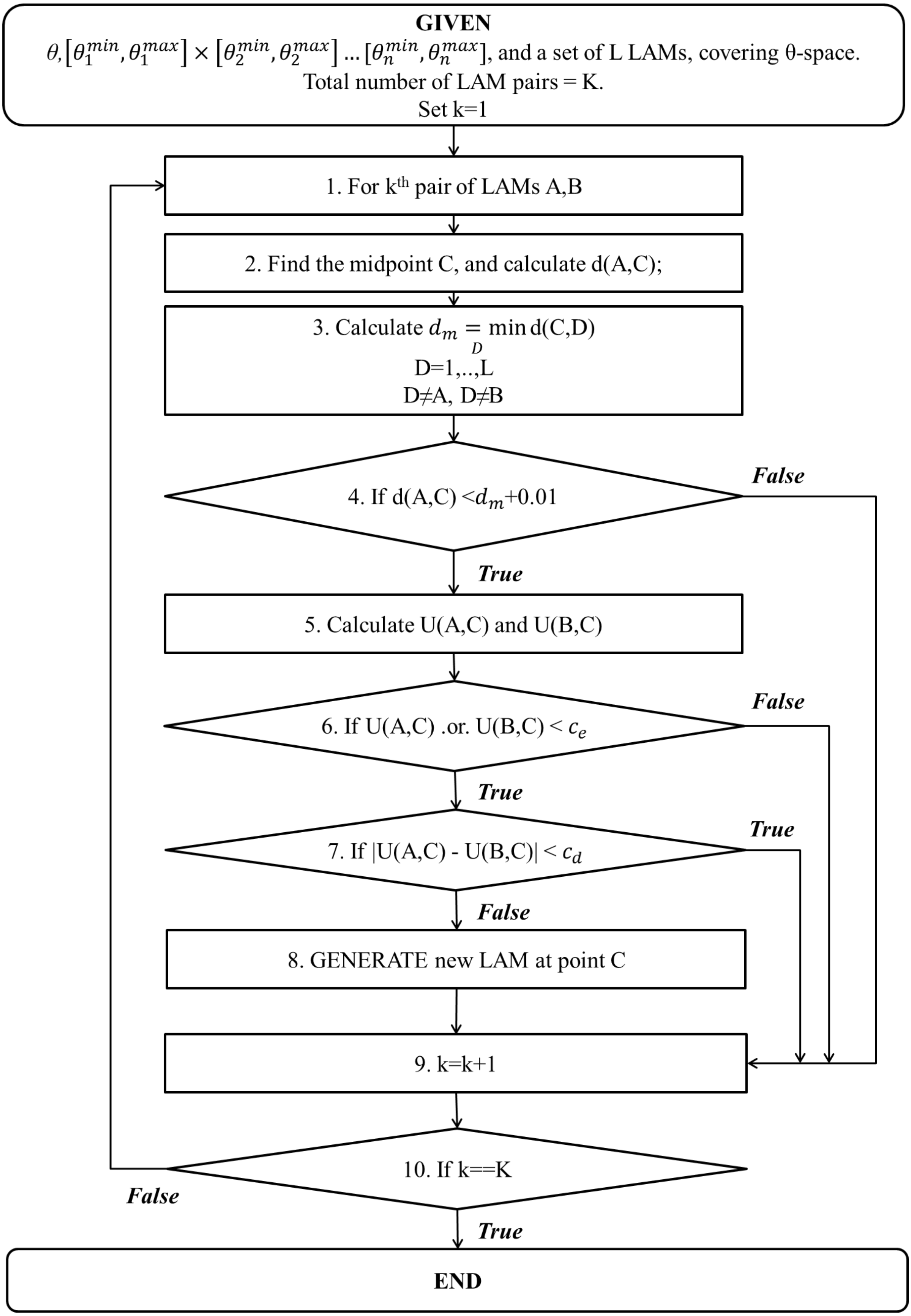

Figure S2 Flowchart of adaptive LAM placement algorithm
